# The complete chloroplast genome of *Triosteum himalayanum* (Caprifoliaceae), a perennial alpine herb

**DOI:** 10.1080/23802359.2019.1693289

**Published:** 2019-11-21

**Authors:** Hai Rui Liu, Jie Fang, Mingze Xia, Qingmeng Xiao, Dejun Zhang, Jing Xie, Shilong Chen

**Affiliations:** a State Key Laboratory of Plateau Ecology and Agriculture, Qinghai University, Xining, China;; b College of Eco-Environmental Engineering, Qinghai University, Xining, China;; c Key Laboratory of Adaptation and Evolution of Plateau Biota, Northwest Institute of Plateau Biology, Chinese Academy of Sciences, Xining, China

**Keywords:** Caprifoliaceae, complete chloroplast genome, phylogeny, *Triosteum himalayanum*

## Abstract

*Triosteum himalayanum* is a perennial herb which is distributed in the eastern Himalayas, Hengduan Mountains, and central China. The complete chloroplast genome of *T. himalayanum* is studied for the first time, which is 154,579 bp in length and is divided into four regions: two inverted repeat (IRA and IRB) regions of 23,370 bp, a small single copy (SSC) region of 18,682 bp and a large single copy (LSC) region of 89,157 bp. The plastid genome contains 133 genes, including 86 protein-coding genes, 39 tRNA genes, and 8 rRNA genes. The overall CG content in the chloroplast genome of *T. himalayanum* is 38.38%. The phylogenetic analysis on the complete plastome sequence of *T. himalayanum* will help to show the intergeneric diversity of Caprifoliaceae.

*Triosteum himalayanum* Wall. which belongs to the genus *Triosteum* of the family Caprifoliaceae is native to the eastern Himalayas, Hengduan Mountains, and central China (including Tibet, Yunnan, Sichuan, Hubei, Shaanxi, and Henan). This species occupies relatively wet habitats on mountain slopes, coniferous forests, streamside, and grasslands. *T. himalayanum* is a perennial herb (Yang et al. 2011; Liu et al. 2018). *T. himalayanum* has a long history as herbal medicine and has been used to inducing diuresis for removing edema and promote blood flow for regulating menstruation (Li 2009). People pay more attention to the chemical constituents and medicinal value of *T. himalayanum.* In this study, we focus on the chloroplast genome sequences which could be used for phylogenetic studies of Caprifoliaceae.

The fresh leaves of *T. himalayanum* were collected from Lulang, Linzhi city, (Tibet, China; 94°43′34.3″E, 29°41′39.0″N) at the altitude of 3500 meters and the voucher specimens (Chen2014305) are deposited in the Herbarium of Northwest Institute of Plateau Biology, Chinese Academy of Sciences (HNWP, CAS), Xining, Qinghai, China. The genomic DNA was extracted from fresh leaves and assembled by Biomarker Technologies Corporation in Beijing on Illumina MiSeq platform. We obtained about 2.75GB sequence data and annotated the genome sequence using GeSeq (https://chlorobox.mpimp-golm.mpg.de/geseq.html; Kang et al. 2018). The manual correction of annotation was performed with Sequin v.15.50 (Hu et al. 2018). The complete chloroplast genome sequence was submitted to GeneBank (GenBank Accession Number: MN551173).

The complete chloroplast genome of *T. himalayanum* is 154,579 bp in length and is divided into four regions: two inverted repeat (IRA and IRB) regions of 23,370 bp, a small single copy (SSC) region of 18,682 bp and a large single copy (LSC) region of 89,157 bp. The plastid genome contains 133 genes, including 86 protein-coding genes, 39 tRNA genes, and 8 rRNA genes. Apart from 16 genes that occurs in double copies, most of the genes only occurs once. They contain five protein-coding types (*rps12, ndhB, rps7, orf42*, and *ycf2*), seven tRNA types (*trnM-CAU, trnI-CAU, trnL-CAA, trnI-GAU, trnV-GAC, trnR-ACG*, and *trnA-UGC*), and four rRNA types (*rrn16, rrn23, rrn4.5*, and *rrn5*). The overall CG content in the chloroplast genome of *T. himalayanum* is 38.38%. The CG content of the LSC, SSC, and IR region are 36.79, 33.01, and 43.56%, respectively.

To analyze the position relationship between *Triosteum himalayanum* and other species of Caprifoliaceae, we utilized 21 published complete cp genome sequences to establish the maximum likelihood tree. We used MAFFT v7.017 to align the whole cp genome sequences (Katoh and Standley 2013), and then, we constructed the maximum likelihood tree by raxmlGUI 1.5b1(Silvestro and Michalak 2012) using 1000 bootstrap replicates. The result shows that *T. himalayanum* is sister to *Triosteum pinnatifidum* and is closely related to genus *Lonicera* in the phylogenetic tree. The phylogenetic analysis on the complete plastome sequence of *T. himalayanum* will help showthe intergeneric diversity of Caprifoliaceae (Figure 1).

**Figure 1.**
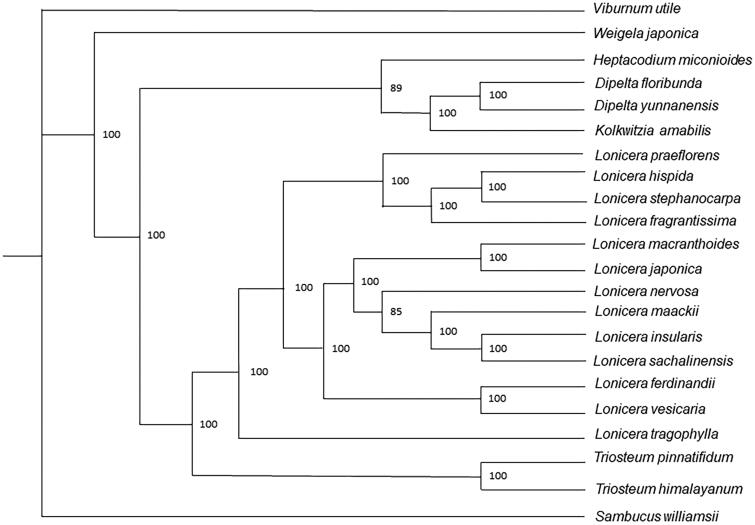
Phylogenetic tree of *Triosteum himalayanum* and other 21 Caprifoliaceae species established by maximum likelihood (ML). Accession numbers: *Dipelta floribunda*, NC_037955.1; *Dipelta yunnanensis*, NC_042201.1; *Heptacodium miconioides*, NC_042739.1; *Kolkwitzia amabilis*, NC_029874.1; *Lonicera ferdinandii*, NC_040963.1; *Lonicera fragrantissima*, MG738669.1; *Lonicera hispida*, NC_040962.1; *Lonicera insularis*, NC_039634.1; *Lonicera japonica*, MH028738.1; *Lonicera maackii*, NC_039636.1; *Lonicera macranthoides*, NC_040959.1; *Lonicera nervosa*, NC_040961.1; *Lonicera praeflorens*, NC_039635.1; *Lonicera sachalinensis*, NC_039637.1; *Lonicera stephanocarpa*, NC_037954.1; *Lonicera tragophylla*, NC_037953.1; *Lonicera vesicaria*, MH028743.1; *Sambucus williamsii*, NC_033878.1; *Triosteum pinnatifidum*, NC_037952.1; *Viburnum utile*, NC_032296.1; *Weigela japonica*, MK397907.1.
